# Comparison of oral dydrogesterone with suppository vaginal progesterone for luteal-phase support in in vitro fertilization (IVF): A randomized clinical trial

**Published:** 2013-11

**Authors:** Saghar Salehpour, Maryam Tamimi, Nasrin Saharkhiz

**Affiliations:** *Infertility and Reproductive Health Research Center (IRHRC), IVF Center, Shahid Beheshti University of Medical Sciences, Tehran, Iran.*

**Keywords:** *In vitro fertilization*, *Luteal-phase support*, *Dydrogesterone*, *Vaginal progesterone*

## Abstract

**Background:** Luteal phase support is mandatory in assisted reproductive technologies (ART) for optimizing outcome, so the luteal phase is supported with either progesterone, addition of estradiol to progesterone, hCG or gonadotropin releasing hormone (GnRH) agonists. Supplementation of luteal phase with progesterone is prescribed for women undergoing routine IVF treatment.

**Objective: **To compare oral dydrogestrone with vaginal progesterone for luteal-phase support in IVF.

**Materials and Methods:** We performed this prospective, randomized trial in a tertiary infertility care unit in Taleghani Hospital, Tehran, Iran. In total 80 Women with a history of male factor infertility undergoing controlled ovarian stimulation for IVF treatment (fresh cycle) randomly were divided in two groups (group A or oral dydrogesterone group and group B or vaginal progesterone group). The inclusion criteria were the use of GnRH analogue down-regulation and age less than 40 years old with regular menstrual cycles. All women were euthyroid and normoprolactinemic. Group A (n=40) received 10 mg dydrogesterone QID (40mg daily) and group B (n=40) received 400 mg suppository vaginal progesterone (cyclogest) twice per day (800 mg daily).

**Results: **Clinical pregnancy rate in cyclogest group was higher than dydrogesterone group but the difference was not significant (p=0.52), furthermore the miscarriage rate in two group was the same .The difference between two groups regarding antral follicle, embryo number, luteal-phase duration, endometrial thickness, oocyte number and metaphase-II was not significant (p>0.05).

**Conclusion:** The results showed that oral dydrogesterone is as effective as vaginal progesterone for luteal-phase support in women undergoing IVF.

## Introduction

It is well established that luteal function is compromised in in vitro fertilization (IVF) cycles and studies on cases undergoing IVF demonstrated that there was a significant reduction in pregnancy rates without luteal-phase support (LPS) (1-3). In the absence of luteal-phase support, the area under the curve for progesterone is suboptimal and accompany by premature luteolysis, short luteal phase and early bleeding (4, 5). Progesterone is necessary for implantation and for the early development of the fertilized ovum. In response to progesterone, the glands become tortuous and secretory and there is an increase in stromal vascularity, thus making the endometrium both morphologically and functionally well prepared for implantation (6). 

In assisted reproductive technologies (ART), luteal phase progesterone supplementation is common practice and several reports concurred that luteal support improves IVF outcome (7-9). Parenteral administration of progesterone, vaginally or I.M, does not subject the compound to the significant metabolic consequences of oral administration. Progesterone administered orally is subjected to first-pass pre-hepatic and hepatic metabolism. This metabolic activity results in progesterone degradation to its 5α and 5β reduced metabolites (10). Dydrogesterone is an optical isomer of progesterone in which the methyl group in carbon 10 is located in α position instead of β position in natural progesterone (11).

These changes in formulation make dydrogesterone more stable and effective orally and it is proved that dydrogesterone has excellent patient compliance, low local adverse effects and ongoing pregnancy rate of 31% after IVF (12). Oral administration is the easiest route of administration, and generally the most acceptable route for the patient. Vaginal administration results in higher uterine concentrations, but is often uncomfortable in the presence of vaginal bleeding, or may be washed out if bleeding is severe. 

Dydrogesterone has a good safety and tolerability profile. It is structurally and pharmacologically similar to natural progesterone, has good oral bioavailability and few side effects. Dydrogesterone has no androgenic effects on the fetus, and does not inhibit the formation of progesterone in the placenta. The medication seemed to have no side effects on the mother. Only Pelinescu-Onciul’s reported drowsiness. Gelle and Schaeffer reported nausea and vomiting, but in only one patient, and Chang, reported nausea and vomiting in two patients. However, nausea and vomiting may be due to early pregnancy itself rather than the medication (13). 

Dydrogesterone seemed to be associated with a higher birth weight, higher 1-min Apgar scores, and a lower incidence of growth retardation. However, these differences were not significant. There seemed to be very few birth defects. Many papers specifically reported no congenital anomalies (13). Other researchers reported potential links between maternal dydrogesterone use during pregnancy and congenital birth defects. The types of defects were very diverse, with no evidence of a pattern of abnormalities. The data do not provide evidence for congenital malformations associated with dydrogesterone use (14).

However, there are limited reports on the use of dydrogesterone in ART cycles for luteal supplementation and these studies have prepared conflicting information about the administration route of progesterone. Patki *et al* indicated that the pregnancy rate is significantly higher with dydrogesterone than with micronized vaginal progesterone and placebo (15). Conversely Levine *et al* compared the pharmacokinetics of an oral micronized progesterone preparation with that of a vaginal progesterone gel and showed that the vaginal gel was associated with a higher maximum serum concentration of progesterone. They concluded that the vaginal administration of progesterone results in a greater bioavailability with less relative variability than oral progesterone (16).

The objective of this study was to compare oral dydrogestrone with natural vaginal progesterone for luteal phase support in IVF. 

## Materials and methods

We directed this prospective, randomized single-blind trial in a tertiary infertility care unit from May to December 2012 in Taleghani Hospital, Tehran, Iran. The study was approved by ethical committee of Shahid Beheshti University of Medical Sciences. The study protocol was explained for all patients and informed written consents were given. In total 80 women with a history of male factor infertility undergoing controlled ovarian stimulation for IVF treatment (fresh cycle) were included in this study. The inclusion criteria were the use of GnRH analogue down-regulation and age less than 40 years old with regular menstrual cycles. All women were euthyroid and normoprolactinemic. 

Women with tubal factor, idiopathic infertility, endometriosis-related infertility, and ovulatory disturbances, moreover, women with baseline FSH >12 IU and adenomyosis, polysyctic ovary, endometriosis, myoma and chronic hepatorenal disease were excluded. All women received a daily subcutaneous (SC) injection of 500 μg GnRH agonist, (Buserelin Suprefact; Aventispharma; Germany), followed by recombinant FSH, 150-300 IU (Gonal-F; Serono; Aubonne, Switzerland) or FSH highly purified (Fostimon; IBSA; Lugano).

Ovarian follicular development was monitored by transvaginal ultrasonography, and 10000 IU human chorionic gonadotrophin (Choriomon; IBSA; Lugano) was administered IM when at least two or more leading follicles reached 18 mm in diameter. Oocytes were retrieved transvaginally under ultrasound guidance 34-36 hours after hCG injection. After egg collection ICSI process was performed. An average of three embryos was transferred 48 to 72 hours after insemination. Luteal-phase support began on the day of oocyte retrieval.

Patients randomly were divided in two groups (group A or oral dydrogesterone group and group B or vaginal cyclogest group). for randomization; numbered sealed envelopes were prepared and provided by the study coordinator, according to random-number tables. Group A (n=40) received 10 mg dydrogesterone QID (Duphaston; Abbot; Istanbul) and group B (n=40) received 400 mg vaginal progesterone twice per day (Cyclogest; Actavis; Barnstaple; UK). The serum β-hCG level was measured 12 days after ET. 

Luteal-phase support was continued up to 12 weeks of pregnancy. Outcome in the two groups was evaluated in terms of clinical pregnancy and miscarriage rates. Clinical pregnancy was defined when an ultrasound scan, performed 6 weeks after ET, revealed the presence of a viable fetus. Miscarriage is the loss of a fetus before the 20^th^ week of pregnancy. The presence of at least one viable fetus at 12 weeks’ gestation was classified as ongoing pregnancy.

**Statistical analysis**

Data were analyzed using SPSS version 20. Categorical data are presented as numbers (%), and continuous data as mean±SD. We used the Chai square (X^2^) or Fisher’s exact test to compare categorical variables and the Student’s *t*-test, to compare continuous variables in two groups. (p≤0.05 is significant)

## Results

There were 82 patients who met the inclusion criteria and were randomly assigned to two groups. Some patients withdrew consent from the study (flowchart of patient participation), therefore for analysis; there were 40 patients in each group who continued participation. No differences between the groups were found in terms of mean age, body mass index and FSH level. This demographic data, including mean age, BMI, and FSH of women in two groups are summarized in table I. The difference between two groups regarding age, BMI and FSH was not significant (p>0.05) [p of Age: 0.13, BMI: 0.98, FSH: 0.83].

Meanwhile, antral follicle, embryo number, lutheal-phase duration, endometrial thickness on the ET day, oocyte number and metaphase-II was similar between two groups (Table II). The difference between two groups regarding (cyclogest-duphaston) was not significant (p>0.05) [P value of antral follicle: 0.349, Embryo number: 0.48, Luteal phase duration: 0.44, Endometrial thickness: 0.21, Oocyte number: 0.59, Metaphase-II: 0.83]. Based on table II, clinical pregnancy rate in cyclogest group was higher than dydrogesterone group but the difference was not significant (p=0.52), furthermore the miscarriage rate in two groups was the same [p=0.95] (Table II). However bleeding and other complications such as nausea and epigastric pain in dydrogestrone group was more than cyclogest group and the difference between two groups was significant (p=0.03 and p=0.009 respectively) (Table II).

**Table I:** Baseline patients characteristics

**Groups**	**Dydrogesterone (N=40)**	**Cyclogest (N=40)**	**p-value**
Mean age, years (SD)	29.4 ± 5.26	31.84 ± 6.10	0.13
Mean BMI, kg/m^2 ^(SD)	24.20 ± 3.04	24.24 ± 3.89	0.98
FSH day 3 (IU/L)	6.85 ± 2.43	7.00 ± 2.42	0.83

BMI: Body Mass Index

**Table II:** Characteristics, clinical outcomes and side effects of drugs in two groups

**Groups**		**Dydrogestrone (N=40)**	**Cyclogest (N=40)**	**p-value**
Antral follicle				0.349
	>7	38 (95%)	34 (85%)	
	<7	2 (5%)	6 (15%)	
Luteal Phase duration (day)				0.44
	>11	23 (63.2%)	26 (69)%	
	<11	15 (37.5%)	10 (25.0%)	
Endometrial thickness		9.08 ± 1.99	8.52 ± 1.15	0.21
Oocyte number		8.44 ± 4.37	9.20 ± 5.47	0.59
Metaphase-II		6.37 ± 3.34	6.60 ± 4.13	0.83
Embryo number		0.63 ± 0.30	0.70 ± 0.31	0.48
Clinical outcomes				
	Pregnant	10 (25%)	13 (32.5%)	0.52
	Miscarriage	3 (7.5%)	3 (7.7%)	0.95
Side effects				
	Bleeding	19 (48%)	8 (20%)	0.03
	Nausea	10 (25%)	0	0.009
	Epigastric pain	6 (15%)	0	0.008

Data presented as mean ±SD or percentage (number).

Chi square (X^2^) test, independent *t* test

**Figure 1:**
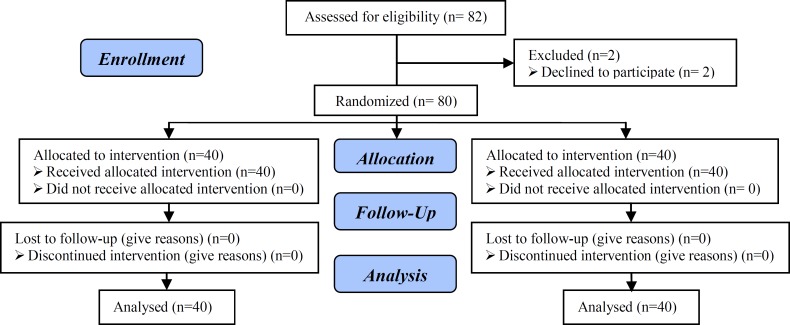
Consort flow diagram

## Discussion

Hormonal support of the luteal phase in assisted reproductive technologies (ART) has historically been an important issue among the researchers (17, 18). Recently, progesterone (P) supplementation has improved outcomes during ART and has been the preferred treatment (17-19). Regarding the administration route of progesterone, intramuscular and transvaginal routes are the two conventional progesterone administration techniques (20). However, very few studies have compared the advantages of oral dydrogestrone with vaginal progesterone for luteal support in ART cycles. 

Dydrogestrone is a retroprogesterone with good oral bioavailability that has a biological active metabolite of progesterone, which has an anti-estrogenic effect on the endometrium producing a secretory transformation (20-23). Vaisbuch *et al* compared the clinical practice for luteal-phase supplementation (LPS) in stimulated IVF cycles in 35 countries, representing a total of 51,155 IVF cycles/year. Vaginal progesterone alone was used for LPS in 64% of cycles and in another 16% of cycles in combination with either i.m. (15%) or oral progesterone (1%). As a single agent, i.m. progesterone was used in 13% of cycles, oral progesterone in another 2% and human chorionic gonadotrophin (HCG) was still used in 5% of cycles (21). 

In this randomized clinical trial, we compared the clinical efficacy of oral dydrogestrone with vaginal progesterone (cyclogest) for LPS in stimulated IVF cycles in 80 women. Regarding demographic data such as age, BMI and FSH on day 3, two groups were properly matched and the difference between them was not significant (p>0.05). Our results showed the clinical pregnancy rate in cyclogest group was higher than dydrogestrone group (32.5% vs. 25%) but the difference was not significant (p=0.52), furthermore the miscarriage rate in two group was the same.

In line with our results a study on LPS in women undergoes IVF by Chakravarty *et al* indicated no significant differences in pregnancy rates, miscarriage rates, or viable delivery rates between women receiving oral dydrogestrone and vaginal micronized progesterone (22). Moreover another randomized clinical trial by Ganesh *et al* supported our results. They compared oral dydrogestrone with progesterone gel and micronized progesterone for luteal-phase support and indicated no significant difference among three groups of women regarding the overall pregnancy and miscarriage rate (23). Additionally, other researchers reported comparable findings to our trial and designated similar efficiency with dydrogestrone and natural micronized progesterone in women undergoing IVF-ET (24-26). 

In present trial the difference between two groups regarding endometrial thickness and FSH level was not significant, conversely, Fatemi *et al *in their trial compared dydrogestrone and natural micronized progesterone in patients with premature ovarian failure and specified significant difference regarding development of endometrial glands, serum progesterone value, LH value and FSH value (27). We designated oral dydrogestrone is as effective as cyclogest for LPS in women undergoing IVF, however bleeding and other complications such as nausea and epigastric pain in dydrogestrone group was more than cyclogest group and the difference between two groups was significant. 

The results of some studies which were reviewed in this article exposed numerous potential benefits of dydrogestrone that proved this agent may be considered as an alternative to vaginal progesterone for LPS. According with these findings we showed no significant difference regarding antral follicle, embryo number, lutheal-phase duration, endometrial thickness, oocyte number and metaphase-II follicles between two groups (p>0.05). Moreover, Ganesh *et al* suggest dydrogestrone is a capable agent for LPS in IVF, moreover the side effects, such as discharge and vaginal irritation, possibly avoided (23). The main limitation of our study was the relatively small sample size. Further investigations are recommended with longer follow-up and larger series to validate the findings reported here.

## Conclusion

In general we confirmed the results of previous reports and showed that oral dydrogestrone is as effective as vaginal progesterone for luteal-phase support in woman undergoing IVF.
